# Relationship between the blood urea nitrogen to serum albumin ratio and in-hospital mortality in patients with type 2 diabetes mellitus complicated with ischemic stroke

**DOI:** 10.1371/journal.pone.0330168

**Published:** 2025-09-10

**Authors:** Peng Wang, Shuyuan Jiang, Yunqi Hua, Wei Xie, Guo Shao, Hongwei Zhu

**Affiliations:** 1 The Second Affiliated Hospital of Baotou Medical College, Inner Mongolia, Baotou, China; 2 School of Pharmacy, Baotou Medical College, Baotou, China; 3 Department of Medical Oncology, Baotou Cancer Hospital, Baotou, China; 4 Inner Mongolia Key Laboratory of Hypoxic Translational Medicine, Baotou Medical College, Inner Mongolia, Baotou, China; 5 Center for Translational Medicine, the Third People’s Hospital of Longgang District, Shenzhen, China; Hormozgan University of Medical Sciences, IRAN, ISLAMIC REPUBLIC OF

## Abstract

**Background:**

Type 2 diabetes mellitus (T2DM) complicated with ischemic stroke is a major challenge to global public health and is related to poor prognosis. However, the role of blood urea nitrogen(BUN)to serum albumin ratio (BAR) in predicting in-hospital mortality of T2DM patients with ischemic stroke has not been fully explored. This study was carried out to investigate the relationship between BAR level and in-hospital mortality of T2DM patients with ischemic stroke.

**Methods:**

The MIMIC-IV database was searched for data on T2DM patients with ischemic stroke. The primary outcome was in-hospital mortality. The BAR was calculated as follows: BUN (mg/dl)/ serum albumin (g/dl). Logistic regression was employed to investigate the relationship between BAR and in-hospital mortality of T2DM patients with ischemic stroke. The restricted cubic spline (RCS) was leveraged to examine the dose-response relationship of BAR with the outcome. The receiver operating characteristic (ROC) curve was utilized to measure the ability of BAR to predict the outcome. In addition, the decision curve analysis (DCA) was employed to explore the value of BAR in clinical practice. The consistency and robustness of the research results were assessed by subgroup analysis and the presence of interactions using a likelihood ratio test.

**Results:**

Finally, 1136 patients were included for evaluation in this study. As BAR levels increased, the in-hospital mortality of T2DM patients with ischemic stroke also increased (OR:1.06; 95% CI:1.01–1.11; P < 0.01). The RCS analysis suggested that there was a linear relationship between BAR and in-hospital mortality of T2DM patients with ischemic stroke (p = 0.276). The ROC curve indicated that BAR was superior to BUN, Sepsis-related Organ Failure Assessment (SOFA), and Glasgow Coma Scale (GCS) in predicting the in-hospital mortality of T2DM patients with ischemic stroke. The DCA curve indicated that the net benefit of BAR was better than BUN, SOFA, and GCS. Subgroup analysis showed that there was no interaction between BAR and each subgroup (all p-value > 0.05).

**Conclusion:**

The in-hospital mortality of T2DM patients with ischemic stroke increased with elevated BAR levels.

## Introduction

Diabetes is a chronic metabolic disease, and its incidence is related to heredity, lifestyle (such as unhealthy diet and lack of exercise), and environmental factors [1]. There is a close relationship between diabetes and ischemic stroke, and about one-third of stroke patients were found to have T2DM [2,3]. Compared to ischemic stroke patients without diabetes, those with diabetes had a higher disability rate, longer hospitalization stays, higher expenses, higher recurrence rates, and increased mortality [4,5]. Therefore, it is very important to early identify high-mortality T2DM patients with ischemic stroke in the intensive care unit (ICU) and selection of appropriate treatment can reduce the risk and improve prognosis. Several scoring systems have been developed to predict the prognosis of T2DM patients with ischemic stroke [6–9]. However, these systems require the collection of many indicators and are highly complex. The objective of this study was to explore the potential of simpler, more convenient markers to predict prognosis in T2DM patients with ischemic stroke.

Blood urea nitrogen (BUN) is an essential indicator for evaluating patients’ renal function, nutrition, and body fluid status [10]. BUN is an important marker of metabolic diseases and patients’ nutritional status and plays a key role in prognostic assessment and mortality prediction in acute and critical patients [11–13]. Serum albumin is an index that reflects the nutrition status of the human body and also has many physiological features including anti-oxidation and anti-inflammation [14–16]. As a new prognostic biomarker uncovered in recent years [17], BAR was regarded as a recently discovered prognostic biomarker. It is a non-invasive and easily accessible tool for predicting the prognosis of critically ill patients, as it provides a comprehensive response to the nutritional and renal status of the body [18,19]. It has been discovered that BAR has good performance in predicting many diseases such as sepsis, pneumonia, chronic obstructive pulmonary disease, and chronic heart failure [17,20–22]. However, the validity of BAR in predicting the mortality of T2DM patients with ischemic stroke has not been confirmed.

The objective of this study was to examine the correlation between BAR and in-hospital mortality of T2DM patients with ischemic stroke and to assess the predictive capacity of in-hospital mortality in this patient group.

## Materials and methods

### Data source

This is a retrospective cohort study, and research data were all derived from the MIMIC-IV database 2.2 (https://mimic.mit.edu/). Established and maintained by the Laboratory for Computational Physiology at the Massachusetts Institute of Technology, this database has comprehensive medical records of inpatients with admission to Beth Israel Deaconess Medical Center from 2008 to 2019 [23]. These records contain demographics, clinical indicators, vital signs, disease names, treatment measures, and survival rates. The database is public, and the identifier is concealed to protect the privacy of patients, so informed consent or ethical approval is not required in this study. The data extraction of this study was completed by Hongwei Zhu, who participated in the training and examination of the Collaborative Institutional Training Initiative (CITI Program) as required and obtained the database access (record ID: 13158546).

### Study population

**Fig 1:**
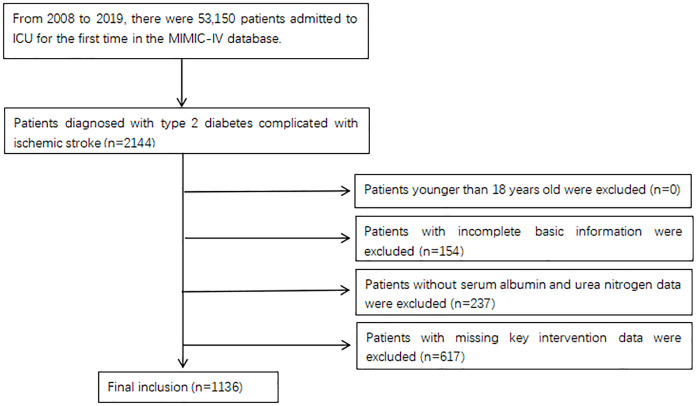
Flowchart of literature screening.

In the MIMIC-IV database, 76,540 patients were admitted to ICU. Among them, 53,150 were admitted for the first time [24]. Inclusion criteria Eligible patients were: (a) those who met the criteria for diagnosing T2DM and ischemic stroke (based on the ninth and tenth editions of the International Classification of Diseases; (b) those who had their first ICU admission; and (c) adults aged ≥18 years. Exclusion criteria: Patients were excluded if they had key variables missing such as albumin, urea nitrogen, main intervention, and treatment indicators. Finally, 1136 T2DM patients with ischemic stroke were included in the analysis (Fig 1 and S1 Table).

### Data extraction

Relevant data were extracted using PostgreSQL 16. The extracted data included: 1) General information of patients: age, gender, race, marital status, and BMI; 2) Laboratory data: blood urea nitrogen, albumin, blood glucose, glycosylated hemoglobin, cholesterol, high-density lipoprotein, low-density lipoprotein, triglyceride, creatinine, white blood cells, red blood cells, and platelets; 3) Disease complications: coronary heart disease, atrial fibrillation, congestive heart failure, hemiplegia, peripheral vascular diseases; 4) Vital signs at first admission: heart rate, blood pressure, respiration, body temperature, SPO2; 5) Clinical score: Acute physiology score III (APSIII), Simplified acute physiological score II (SAPSII), SOFA, GCS score; 6) Medications: aspirin, digoxin, statins, norepinephrine, vancomycin, insulin; and 7) Invasive operation and examination: mechanical ventilation and dialysis. BAR (mg/g) was calculated as follows: BUN (mg/dl)/ serum albumin (g/dl).

### Handling of outliers and missing values

The researchers first deleted variables with missing values greater than 20%, such as systolic blood pressure, diastolic blood pressure, BMI, and other variables. Variables with a missing value below 20%, such as cholesterol, triglycerides, high-density lipoprotein, low-density lipoprotein, and glycosylated hemoglobin, were supplemented by multiple completions. This process was completed by the “mice” package in R software, and the random forest algorithm (trained by other non-missing variables) was used for multiple substitution processing. The cap method was adopted to deal with the variables with abnormal values, with a cutoff of 1 and 99%.

### Outcome

The primary endpoint of this study was defined as in-hospital all-cause mortality. The outcome was the in-hospital mortality, which is determined as the survival state at discharge. The follow-up time was set at a range from admission to discharge or death. Patients without any recorded results or follow-up time were finally ruled out.

### Statistical analysis

The continuous variables of normal distribution were represented by the mean ± standard deviation, and the continuous variables of non-normal distribution were presented by the median and its quartile interval. For data of normal distribution, analysis of variance (ANOVA) was employed, while for those deviating from normal distribution, the Kruskal-Wallis test was adopted. Categorical variables were expressed in total and percentage and compared by chi-square test.

Three logistic regression models were employed to explore the relationship between BAR and in-hospital mortality of T2DM patients with ischemic stroke and generated odds ratio (OR) and 95% confidence interval. BAR tertile was used as a reference and to analyze the BAR as a continuous and categorical variable. Details of the model were as follows: model 1 (unadjusted), model 2 (adjusted for age, sex, race and marital status) and model 3 (adjusted for age, sex, race, marital status, heart rate, respiration, total cholesterol, creatinine, blood glucose, glycosylated hemoglobin, red blood cells, triglycerides, white blood cells, blood oxygen saturation, atrial fibrillation, coronary heart disease, heart failure, aspirin, norepinephrine, vancomycin, and mechanical ventilation). The RCS model was utilized to examine the dose-response relationship of BAR with all-cause mortality in T2DM patients with ischemic stroke. ROC analysis was utilized to investigate the strength of BAR, BUN, SOFA, APSIII, and SAPSII in predicting in-hospital mortality, and the area under the curve (AUC) was also calculated. The clinical application value of BAR was explored by decision curve analysis (DCA). Finally, the influence of general data, complications, and main intervention measures on the research results was further analyzed by subgroup analysis, which aimed at evaluating the reliability and robustness of the research results. Meanwhile, the interaction between BAR and subgroups was assessed by the likelihood ratio test. R software 4.3.2 was utilized for data analysis. A p-value of lower than 0.05 (two-tailed) was deemed as having statistical significance.

## Results

### Baseline characteristics

A total of 1136 T2DM patients with ischemic stroke were included in this study (Fig 1). Patients were classified into three groups according to the BAR tertile (T1: ≤ 4.40; T2: 4.40–6.97; T3: > 6.97), and the baseline characteristics of the three groups are provided in Table 1. A higher BAR was usually associated with old age, a greater proportion of patients with heart failure, coronary heart disease, and atrial fibrillation, and an increased use of vasoactive agents, antibiotics, and mechanical ventilation. In addition, the scores of APSIII, SAPSII, SOFA, and GCS in patients with greater BAR were all high. The levels of blood urea nitrogen, creatinine, white blood cells, and red blood cells were higher in patients with high BAR than in patients with low BAR, while the levels of albumin, cholesterol, high-density lipoprotein, low-density lipoprotein, and platelet were lower in patients with higher BAR levels. Differences in the values of blood glucose, glycosylated hemoglobin, and triglyceride were not noted among the groups. With the increase in BAR, the in-hospital mortality of T2DM patients with ischemic stroke also went up.

**Table 1:** Baseline characteristics and outcomes of patients by BAR.

	Overall (N = 1136)	T1(N = 379)	T2(N = 379)	T3(N = 378)	*p*.value
BAR	7.21 ± 5.78	3.34 ± 0.70	5.51 ± 0.76	12.8 ± 7.09	<0.001
Demographic variables					
age	70.0 (61.0,79.0)	66.0 (56.0,74.0)	71.0 (62.0,79.0)	73.0 (65.0,82.0)	<0.001
Gender:					0.596
Male	588 (51.8%)	194 (51.2%)	204 (53.8%)	190 (50.3%)	
Female	548 (48.2%)	185 (48.8%)	175 (46.2%)	188 (49.7%)	
Marital status:					0.03
Single	311 (27.4%)	117 (30.9%)	93 (24.5%)	101 (26.7%)	
Married	559 (49.2%)	181 (47.8%)	206 (54.4%)	172 (45.5%)	
Other	266 (23.4%)	81 (21.4%)	80 (21.1%)	105 (27.8%)	
Race:					<0.001
White	698 (61.4%)	200 (52.8%)	259 (68.3%)	239 (63.2%)	
Other	438 (38.6%)	179 (47.2%)	120 (31.7%)	139 (36.8%)	
Vital signs					
HR (bpm)	98.2 (97.7,98.8)	98.3 (97.8,98.8)	98.2 (97.7,98.7)	98.1 (97.5,98.8)	0.001
RR(bpm)	18.0 (15.0,22.0)	18.0 (15.0,22.0)	18.0 (15.0,22.0)	19.0 (16.0,22.0)	0.128
SpO2 (%)	85.0 (85.0,88.0)	85.0 (85.0,88.0)	85.0 (85.0,88.0)	85.0 (85.0,88.0)	0.01
Laboratory Parameters					
ALB (g/dL)	3.90 (3.40,4.20)	4.10 (3.80,4.40)	3.90 (3.50,4.20)	3.50 (3.00,3.90)	<0.001
BUN(mg/dL)	21.0 (15.0,30.0)	13.0 (11.0,16.0)	21.0 (18.0,24.0)	36.0 (30.0,51.0)	<0.001
TC(mg/dL)	157 (130,195)	168 (140,210)	158 (130,189)	148 (119,180)	<0.001
Cr(mg/dL)	1.10 (0.80,1.40)	0.80 (0.70,1.00)	1.00 (0.80,1.20)	1.60 (1.20,2.40)	<0.001
FBG (mg/dL)	152 (114,212)	154 (116,218)	145 (112,196)	158 (117,228)	0.082
HBA1C(%)	6.90 (6.20,8.10)	6.90 (6.20,8.30)	6.90 (6.20,8.00)	6.90 (6.10,8.10)	0.374
HDL (mg/dL)	43.0 (34.0,54.0)	46.0 (36.0,55.0)	43.0 (34.0,54.0)	41.0 (32.0,54.0)	0.006
LDL (mg/dL)	81.0 (59.0,114)	93.0 (69.0,126)	81.0 (58.0,108)	74.5 (52.0,103)	<0.001
TG (mg/dL)	129 (92.0,186)	126 (92.0,192)	132 (92.5,183)	129 (91.0,186)	0.938
WBC(K/uL)	8.55 (6.80,11.0)	8.30 (6.60,10.4)	8.60 (6.90,10.8)	8.80 (6.90,12.1)	0.008
RBC(m/uL)	4.30 (3.81,4.76)	4.57 (4.18,4.89)	4.37 (3.94,4.80)	3.95 (3.46,4.41)	<0.001
Platelets(K/uL)	239 (188,291)	248 (197,292)	242 (190,292)	226 (177,287)	0.004
Comorbidity diseases, n (%)					
AF:					<0.001
No	601 (52.9%)	232 (61.2%)	194 (51.2%)	175 (46.3%)	
Yes	535 (47.1%)	147 (38.8%)	185 (48.8%)	203 (53.7%)	
CVD:					0.002
No	712 (62.7%)	264 (69.7%)	227 (59.9%)	221 (58.5%)	
Yes	424 (37.3%)	115 (30.3%)	152 (40.1%)	157 (41.5%)	
Congestive heart failure					<0.001
No	790 (69.5%)	298 (78.6%)	269 (71.0%)	223 (59.0%)	
Yes	346 (30.5%)	81 (21.4%)	110 (29.0%)	155 (41.0%)	
CKD:					<0.001
No	597 (52.6%)	281 (74.1%)	207 (54.6%)	109 (28.8%)	
Yes	539 (47.4%)	98 (25.9%)	172 (45.4%)	269 (71.2%)	
Interventions, n (%)					
Aspirin:					0.049
No	110 (9.68%)	48 (12.7%)	29 (7.65%)	33 (8.73%)	
Yes	1026 (90.3%)	331 (87.3%)	350 (92.3%)	345 (91.3%)	
Norepinephrine:					<0.001
No	815 (71.7%)	300 (79.2%)	279 (73.6%)	236 (62.4%)	
Yes	321 (28.3%)	79 (20.8%)	100 (26.4%)	142 (37.6%)	
Vancomycin:					0.002
No	554 (48.8%)	201 (53.0%)	197 (52.0%)	156 (41.3%)	
Yes	582 (51.2%)	178 (47.0%)	182 (48.0%)	222 (58.7%)	
ACEI:					0.746
No	477 (42.0%)	165 (43.5%)	158 (41.7%)	154 (40.8%)	
Yes	658 (58.0%)	214 (56.5%)	221 (58.3%)	223 (59.2%)	
Mechanical ventilation					0.022
No	520 (45.8%)	191 (50.4%)	176 (46.4%)	153 (40.5%)	
Yes	616 (54.2%)	188 (49.6%)	203 (53.6%)	225 (59.5%)	
Clinical severity					
APSIII	42.0 (32.0,55.0)	35.0 (26.0,46.0)	40.0 (31.0,52.0)	51.0 (41.0,65.0)	<0.001
GCS	15.0 (14.0,15.0)	15.0 (14.0,15.0)	15.0 (15.0,15.0)	15.0 (14.0,15.0)	0.005
SAPSII	35.0 (28.0,43.0)	30.0 (25.0,37.0)	34.0 (27.0,42.0)	40.0 (33.0,50.0)	<0.001
SOFA	1.00 (0.00,3.00)	1.00 (0.00,2.00)	1.00 (0.00,2.00)	1.50 (0.00,4.00)	<0.001
Outcomes, n (%)					
Status:					0.001
No	1081 (95.2%)	367 (96.8%)	367 (96.8%)	347 (91.8%)	
Yes	55 (4.84%)	12 (3.17%)	12 (3.17%)	31 (8.20%)	

Skewed data were represented by mean ± standard deviation or median (IQR), and the data of categorical variables were represented by percentages. BAR grouping: T1: ≤ 4.40; T2:4.40–6.97; T3: > 6.97; HR, heart rate; RR, respiratory frequency; BUN, blood urea nitrogen; ALB, serum albumin; TC, total cholesterol; Cr, creatinine; WBC, white blood cells; RBC, red blood cells; PLT, Platelets; FBG, fasting blood glucose; HBA1C, glycosylated hemoglobin; HDL, high-density lipoprotein; LDL, low-density lipoprotein; TG, triglyceride; AF, atrial fibrillation; APSIII, acute physiological score III; SAPSII, simplified acute physiological score II; GCS, glasgow coma scale; SOFA score, sequential organ failure assessment score; ACEI, angiotensin-converting enzyme inhibitor; CKD, chronic kidney disease.

### Correlation between BAR and prognosis in T2DM patients with ischemic stroke

We established three logistic regression models to investigate the predictive performance of BAR for the mortality of T2DM patients with ischemic stroke. The model suggested that: BAR was significantly correlated with in-hospital mortality in the unadjusted model when it was considered as a continuous variable (OR: 1.07; 95% CI: 1.04–1.10; P < 0.001), indicating its capability to predict in-hospital mortality. In addition to BUN and albumin, the influence of confounding factors was gradually considered in logistic regression. After the variables such as age, gender, race, marital status, heart rate, respiration, total cholesterol, creatinine, blood glucose, glycosylated hemoglobin, red blood cells, triglycerides, white blood cells, oxygen saturation, atrial fibrillation, CVD, heart failure, CKD, aspirin, ACEI, norepinephrine, vancomycin, and mechanical ventilation all entered the adjusted multivariate logistic regression model, BAR was still significantly correlated with the in-hospital mortality (OR:1.06; 95% CI:1.01–1.11; P < 0.01). Besides, when BAR was used as a categorical variable, the mortality risk of BART3 was significantly higher than that of BART1 in the unadjusted model, and a significant difference still existed in the fully adjusted model (T1 and T3: OR:3.458; 95% CI:1.366–9.414; P < 0.01). The results of binary logistic regression analysis are displayed in Table 2.

**Table 2:** Logistic regression analysis of BAR and in-hospital mortality of T2DM patients with ischemic stroke.

Variable	Model 1			Model 2			Model 3		
	OR	OR 95% CI	*P*-value	OR	OR 95% CI	*P*-value	OR	OR 95% CI	*P*-value
BAR	1.07	1.04-1.10	<0.001	1.07	1.038-1.102	<0.001	1.06	1.01-1.11	<0.05
BAR									
T1	Reference			Reference			Reference		
T2	1	0.44-2.28	1	0.98	0.42-2.25	0.955	1.505	0.566-4.068	0.412
T3	2.73	1.42-5.61	<0.01	2.59	1.32-5.41	<0.01	3.458	1.366-9.414	<0.01

Model 1: baseline (unadjusted).

Model 2: adjusted taking into account race, gender, age,and marital status.

Model 3: adjusted for multivariate variables: age, race, gender, marital status, HR, RR, SPO2, cholesterol, creatinine, blood glucose, HBA1C, RBC,TG, WBC, AF, CVD, CHF, CKD, aspirin, ACEI, norepinephrine, vancomycin, Mechanical ventilation.

**Fig 2:**
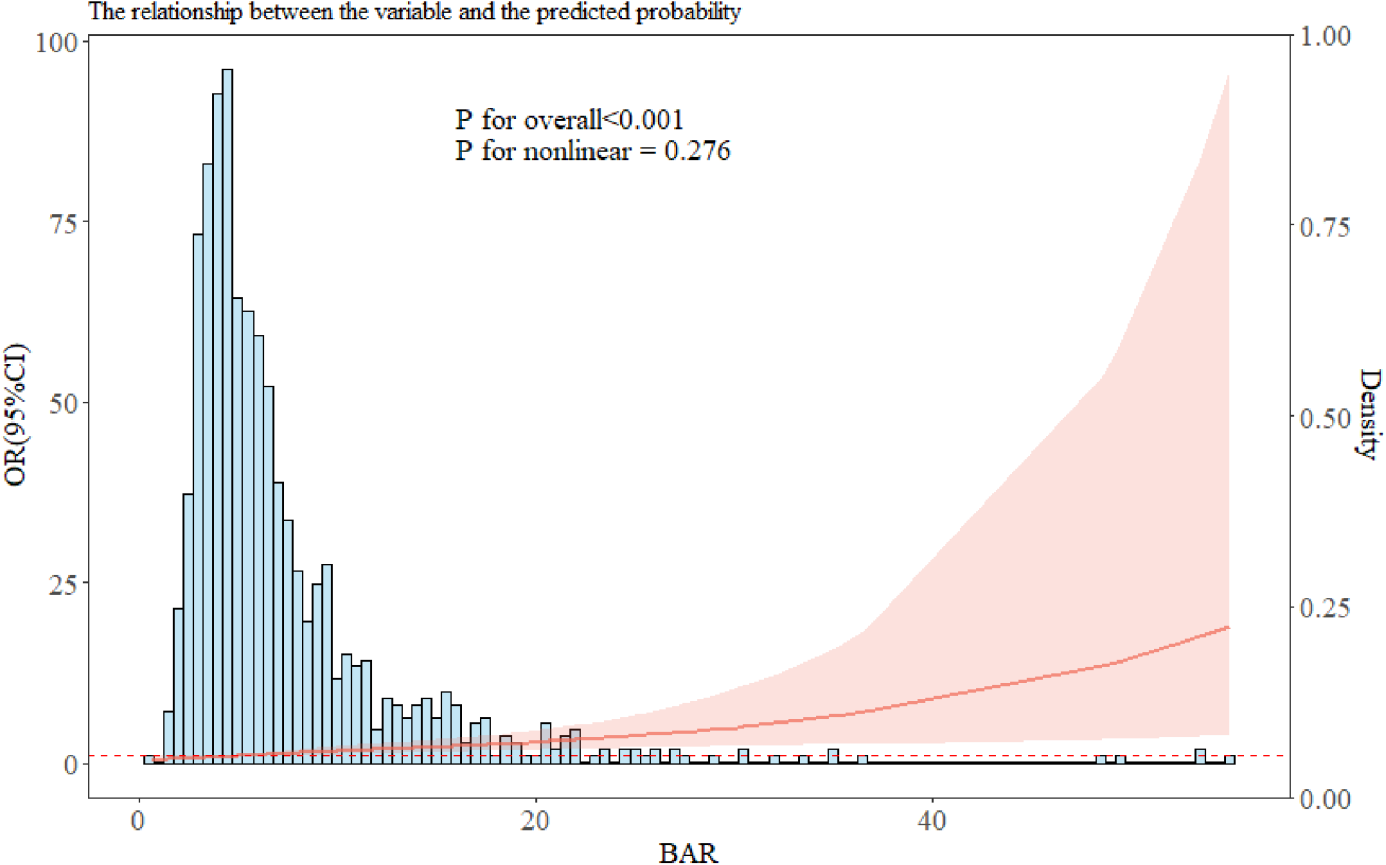
RCS curve of BAR and in-hospital mortality in T2DM patients with ischemic stroke.

The restricted cubic spline model was employed to test the dose-response relationship of BAR with the in-hospital mortality of T2DM patients with ischemic stroke. The results suggested that there was a linear relationship between BAR and the in-hospital mortality of T2DM patients with ischemic stroke (p = 0.276), which further proved that the increase in BAR is linked with a greater risk of in-hospital mortality of T2DM patients with ischemic stroke (Fig 2).

### Prognostic value of BAR in T2DM patients with ischemic stroke

**Fig 3:**
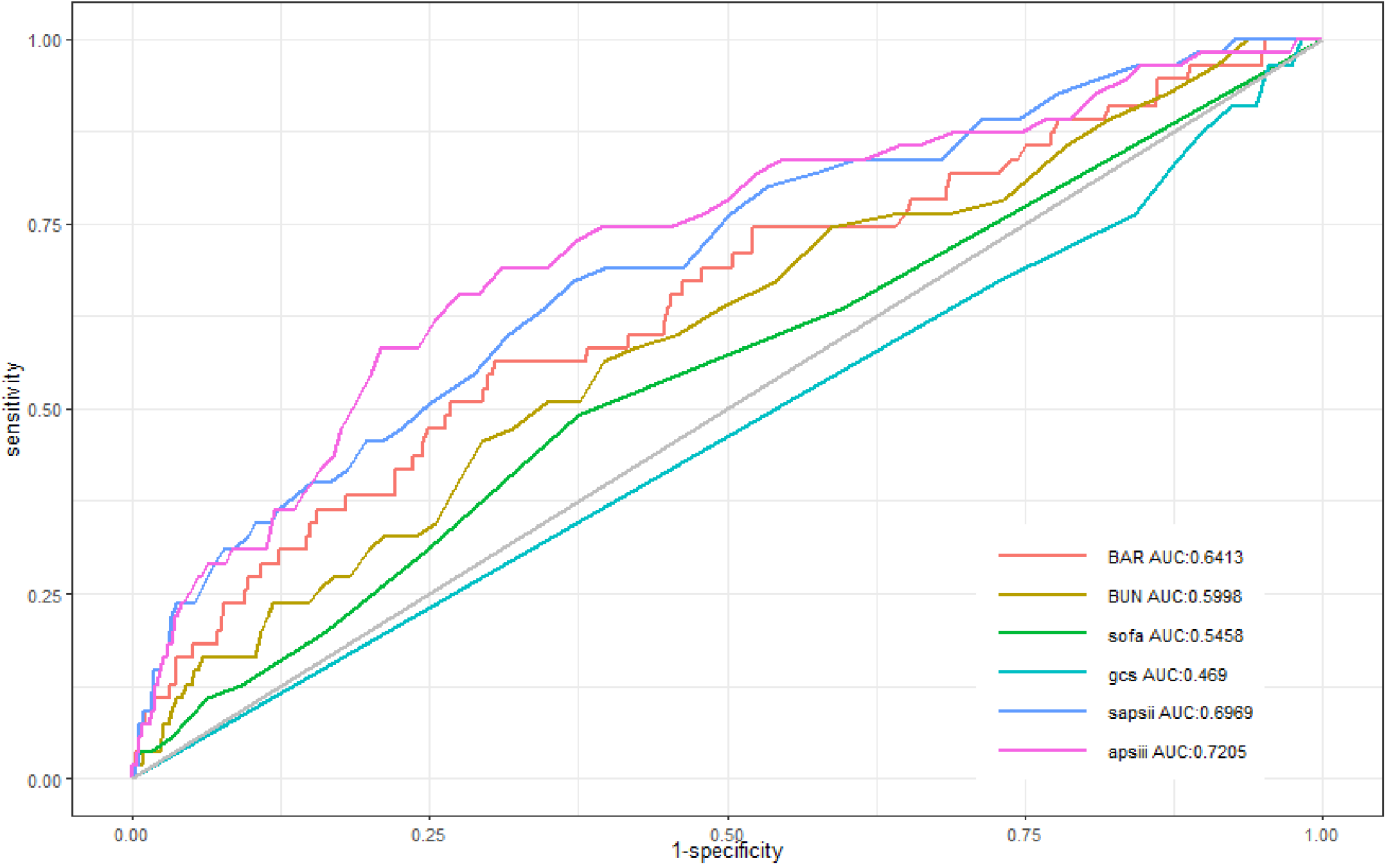
ROC curve.

**Fig 4:**
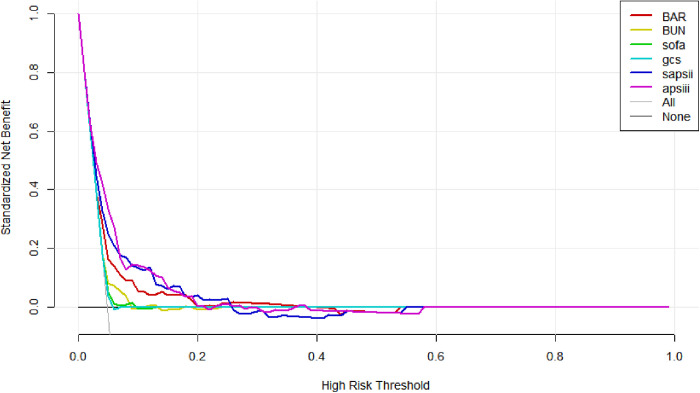
DCA curve.

The ROC curve was used to investigate the predictive strength of BAR in T2DM patients with ischemic stroke and to compare BAR with BUN, SOFA, APSIII, and SAPSII. The AUC areas of BAR, BUN, SOFA, GCS, SAPSII, and APSIII were 0.64, 0.60, 0.55, 0.47, 0.70, and 0.72, respectively, which indicated that BAR was superior to BUN, SOFA, and GCS in predicting the in-hospital mortality of T2DM patients with ischemic stroke. The ROC curve is shown in Fig 3, and the specific parameters of ROC curve analysis are displayed in Table 3. BAR sensitivity and specificity were 0.70 and 0.56, respectively, both higher than BUN. The optimal cutoff value of BAR and BUN was 7.22 mmol/L and 23.5 mg/dL, respectively. When the risk threshold possibility is within 0–100%, the larger area under the decision curve (AUC) indicates the greater net benefit of the corresponding model. As shown in the figure, the net benefit of BAR was greater than that of BUN, SOFA, and GCS, indicating that RAR may have a good clinical effect (Fig 4).

**Table 3:** Comparison of parameters related to the ROC curve.

	AUC	95% CI	Threshold	Specificity	Sensitivity	Youden’s index
BARROC	0.64	0.56-0.72	7.22	0.70	0.56	0.26
BUNROC	0.60	0.52-0.68	23.5	0.60	0.56	0.17
SOFAROC	0.55	0.47-0.62	1.50	0.63	0.49	0.12
GCSROC	0.47	0.40-0.54	5.50	0.02	1.00	0.02
SAPSIIROC	0.70	0.62-0.77	38.5	0.63	0.67	0.30
APSIIIROC	0.72	0.65-0.79	50.5	0.69	0.69	0.38

### Subgroup analysis

**Fig 5:**
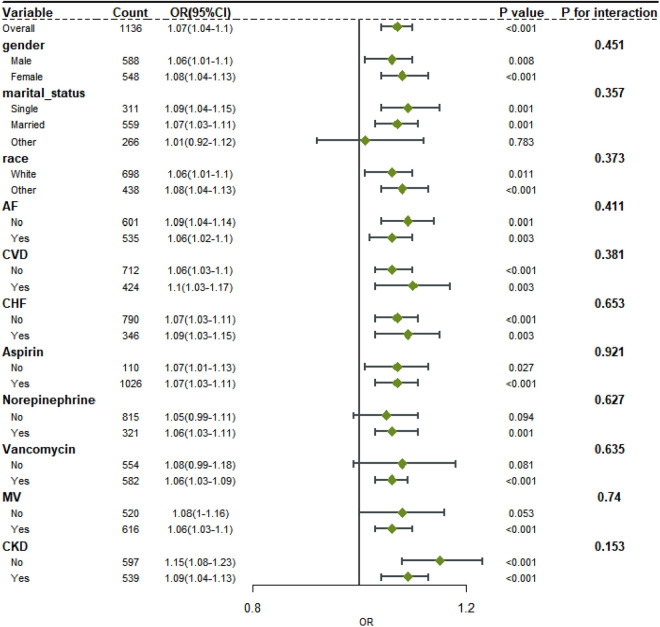
Subgroup analysis of the association between BAR and in-hospital mortality of T2DM patients with ischemic stroke.

Stratification and interaction analysis were carried out according to the patient’s gender, marital status, race, disease complications, and intervention measures. Interaction analysis showed that there was no interaction between BAR and each subgroup (P > 0.05). This indicates that the influence of BAR on the in-hospital mortality of T2DM patients with ischemic stroke was consistent among all subgroups, and BAR is linked with the in-hospital mortality of most subgroups of T2DM patients with ischemic stroke, as shown in Fig 5. Furthermore, S2 Table and S1 Fig provide detailed results of the subgroup analysis comparing patients with no missing data to those with some missing data. The analysis demonstrates no significant differences in outcomes between the two groups, thereby validating the robustness of our imputation methods.

## Discussion

T2DM patients with ischemic stroke have a different clinical pattern and worse prognosis than non-diabetic stroke patients [25]. Scoring systems such as SOFA, GCS, and Acute Physiology And Chronic Health Evaluation II (APACHE II) have shown potential for predicting the prognosis of T2DM patients with ischemic stroke [26]; however, the complexity of calculating these scores is such that not all patients can be screened by the clinician [27]. Therefore, there is an urgent need for a simpler, faster, highly reproducible, and sensitive indicator of the severity of T2DM patients with ischemic stroke. The association of BAR with in-hospital mortality in T2DM patients with ischemic stroke was explored in the current study. At the same time, the role of a high BAR level as an independent predictor of in-hospital mortality in T2DM patients with ischemic stroke in the ICU was demonstrated. Even after adjusting for potential confounding factors, BAR still has an independent association with in-hospital mortality of T2DM patients with ischemic stroke. Therefore, BAR is worthy of attention in clinical practice and can be applied to predict in-hospital mortality in T2DM patients with ischemic stroke independently.

Blood urea nitrogen is a metabolic waste produced by the liver and excreted by the kidney in protein [28,29], which is used as a biomarker to evaluate renal function together with glomerular filtration rate, creatinine, and other indicators [30]. It is well known that the increase in BUN is an important prognostic indicator for patients with end-stage heart, kidney diseases or some other life-threatening diseases [31]. In some clinical researches, it was found that the level of BUN was higher in cardiovascular disease patients, and there was a positive association with both cardiovascular and all-cause mortality [32,33]. Therefore, the evaluation of BUN is considered an effective predictor of cardiovascular disease prognosis [34,35].

Albumin levels can reflect the nutritional and inflammatory state of the human body [36]. Low serum albumin concentration has been proven to be linked with an elevated incidence and mortality of myocardial infarction, coronary heart disease, stroke, as well as with all-cause mortality, cardiovascular mortality, and cancer mortality [32,37–41]. Hypoalbuminemia is related to a raised risk of cardiovascular diseases in elderly patients [42]. Even within the normal range, patients with decreased serum albumin concentration may be at risk of cardiovascular disease [43].

The ideal biomarker should be easily accessible, inexpensive, non-invasive, and able to reflect the specific pathophysiological mechanisms of the disease [44]. The BAR combines the clinical values of BUN and albumin, considering several factors including hepatic and renal status, protein metabolism, and nutritional status. The simplicity and accessibility of BAR make it an attractive tool for clinicians, especially in settings demanding rapid assessment. At present, it has been proved that it is related to the mortality risk of sepsis [18,20], cardiac surgery [44], pneumonia [45,46], chronic heart failure [17], AKI [46–48], and many other diseases [49,50]. Zhao et al. demonstrated that BAR exhibited a superior predictive capacity for long-term mortality following acute myocardial infarction, outperforming the APSIII and SOFA [24]. Li et al. demonstrated that BAR was more accurate than APACHE IV and SOFA in predicting in-hospital mortality in patients with acute ischemic stroke [51]. Shi et al. showed that BAR was more predictive than SOFA in predicting mortality in AKI patients [47]. Our study has shown that the AUC of BAR was higher than that of SOFA and GCS in predicting the in-hospital mortality of T2DM patients with ischemic stroke, which indicated better predictive performance of BAR than SOFA and GCS for T2DM patients with ischemic stroke. Clinicians may predict the risk of mortality of T2DM patients with ischemic stroke through evaluation of BAR and take more active management and treatment measures at an early stage to improve the prognosis of critically ill patients. However, compared to established scoring systems such as SOFA, GCS, and APACHE II, which have been extensively validated and are widely used in clinical practice for their comprehensive assessment of patient prognosis, BAR remains a novel clinical marker. As such, BAR necessitates further validation through large-scale randomized controlled trials to confirm its utility and to fully elucidate its role in clinical practice.

ROC curve analysis in our study identified a BAR threshold of 7.22. This value is consistent with findings from previous studies. For instance, one study found that the BAR exhibited significant predictive ability for 90-day mortality, with an optimal threshold of 6.587 [52]. Another study reported a BAR threshold of 6.0 as a prognostic predictor of acute kidney injury and in-hospital mortality in ICU patients with intracerebral haemorrhage [50]. These consistent findings across studies suggest that a BAR value ranging from 6.5 to 7.2 mg/g could be a robust indicator of increased mortality risk.

Several limitations were encountered in this study. Firstly, this study is a retrospective study. Although the researchers conducted multivariate adjustment and subgroup analysis, the possibility of residual confounding factors still exists. Secondly, it is impossible to extract the specific onset time of T2DM patients with ischemic stroke and the patient’s specific cause of death in this database. So it is unfeasible to carry out further research. Thirdly, only the results of the first test after the patient was admitted to the ICU were used to calculate the BAR. It is necessary to explore more research on different time points and dynamic change levels. Fourthly, variables with a missing value below 20% were supplemented by multiple imputation methods, which may introduce biases. Finally, our research was conducted in the American population, and it remains unclear whether our findings can be generalized to other populations. Considering these factors, prospective, randomized controlled trials are needed for further validation.

## Conclusion

With the increase of BAR, the in-hospital mortality of T2DM patients with ischemic stroke gradually increased. This indicates that BAR has a superior predictive ability for the in-hospital mortality of T2DM patients with ischemic stroke.

## Supporting information

S1 Fig Subgroup Analysis of Patients with No Missing vs. Patients with Some Missing Data Before Imputation. (DOCX)

S1 Table Original data information for participants. (XLSX)

S2 Table Comparison of Variables Between Groups with No Missing Data and Groups with Some Missing Data. (XLSX)
