# Supplementary figures and images for: Relationship between the blood urea nitrogen to serum albumin ratio and in-hospital mortality in patients with type 2 diabetes mellitus complicated with ischemic stroke
Source: PLoS One. 2025 Sep 10;20(9):e0330168. doi: 10.1371/journal.pone.0330168 (PMC12422443; doi:10.1371/journal.pone.0330168)

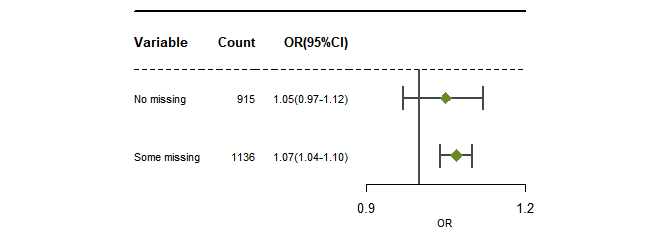


**S1 Fig. Subgroup Analysis of Patients with No Missing vs. Some Missing Data Before Imputation**

Supplement: S1 Fig — (DOCX) [file pone.0330168.s001.docx]
